# Factors associated with married women’s support of male circumcision for HIV prevention in Uganda: a population based cross–sectional study

**DOI:** 10.1186/s12889-016-3385-2

**Published:** 2016-08-02

**Authors:** Komi Mati, Korede K. Adegoke, Hamisu M. Salihu

**Affiliations:** 1 Department of Epidemiology and Biostatistics, University of South Florida, 13201 Bruce B. Downs Blvd. MDC 56, Tampa, FL USA; 2 Department of Family and Community Health, Baylor College of Medicine, Houston, TX USA

**Keywords:** Medical male circumcision, HIV, Women, Sexual bargaining power

## Abstract

**Background:**

Despite the protective effect of male circumcision (MC) against HIV in men, the acceptance of voluntary MC in priority countries for MC scale–up such as Uganda remains limited. This study examined the role of women’s sociodemographic characteristics, knowledge of HIV and sexual bargaining power as determinants of women’s support of male circumcision (MC).

**Methods:**

Data from the Uganda AIDS Indicator Survey, 2011 were analyzed (*n* = 4,874). Bivariate and multivariate logistic regression analyses with random intercept were conducted to identify factors that influence women’s support of MC.

**Results:**

Overall, 67.0 % (*n* = 3,276) of the women in our sample were in support of MC but only 28.0 % had circumcised partners. Women who had the knowledge that circumcision reduces HIV risk were about 6 times as likely to support MC than women who lacked that knowledge [AOR (adjusted odds ratio) = 5.85, 95 % CI (confidence interval) = 4.83–7.10]. The two indicators of women’s sexual bargaining power (i.e., ability to negotiate condom use and ability to refuse sex) were also positively associated with support of MC. Several sociodemographic factors particularly wealth index were also positively associated with women’s support of MC.

**Conclusions:**

The findings in this study will potentially inform intervention strategies to enhance uptake of male circumcision as a strategy to reduce HIV transmission in Uganda.

## Background

There is a growing appeal from the World Health Organization (WHO), the UN Joint Program on HIV/AIDS (UNAIDS) and the President’s Emergency Plan for AIDS Relief (PEPFAR) for quick scale–up of medical male circumcision (MMC) in areas of high HIV prevalence in sub–Saharan Africa [1–3]. The interest of these organizations in MMC follows findings from several studies, which include three randomized control trials that demonstrated a protective effect of MMC against HIV in men [4–6]. Furthermore, a recent epidemiological and economic modeling of the impact and cost of MMC scale-up shows that MMC may be cost-effective [7]. Despite these potential advantages, the acceptance of voluntary MMC in identified priority countries remains limited. Uganda, one of the countries in East Africa that has been identified as a priority country for MMC scale-up, had only reached 11 % of its goal by the end of 2012 [8]. In order to achieve rapid uptake of MMC through targeted interventions in Uganda, it is important to identify factors associated with support for MMC.

Although men are the main focus of MMC education, the literature shows that women can play a central role in the scale-up of MMC [9–13]. Women’s beliefs about MMC and their endorsement of MMC significantly influence their male partners’ or sons’ acceptance of MMC [14, 15]. A recent intervention study found an increase in the likelihood of undergoing MMC among participants in their experimental group and attributed this to increased acceptance of MMC among their female partners [16]. Women play a key role in encouraging men to get circumcised and they may be valuable in strategies for increasing MMC uptake [9, 15, 16]. As a result, it is important to investigate the determinants of support for MMC among females.

Studies suggest that knowledge or belief in the protective effect of circumcision on HIV and STI risk may facilitate MMC acceptability among women [12, 17, 18]. Several sociodemographic factors including educational level and religious affiliation have been linked to support for MMC [12, 18]. Another important factor that is related to women’s support for MMC include the possibility of having sex with a circumcised man without a condom due to lack of perceived HIV risk [12, 19, 20]. Interestingly, women who oppose MMC have also cited the fear of having sex without a condom as their main reason [12]. A qualitative study in Papua New Guinea found that women’s fears about MMC resulting in sexual risk compensation in terms of a decline of condom use was one of the main reasons they didn’t support MMC [20]. According to another study, gender relations might negatively influence women’s support of MMC through their reaction to the risk compensation behavior that circumcised men may adopt [19]. Maughan-Brown, and Venkataramani [19] suggest that women may give up a costly bargaining for safe sexual practice if they believe erroneously that circumcision prevents HIV transmission from men to women as it did in the case of women to men. Though, that may be potentially true in the case of unmarried women, we do not expect that women in a stable union will give up bargaining a protective behavior because of knowledge of a potential decrease in HIV transmission risk due to MMC. We instead hypothesize that married women who have sexual bargaining power may support MMC knowing that they can easily convince their sexual partner after circumcision to use condoms. This is attributable to the increased awareness among these women about STI risk factors including the protective role of condom use.

In this study, we aim to investigate the association between several women’s characteristics including their sexual bargaining power and their support of male circumcision (MC) in Uganda using nationally representative data.

## Methods

### Data and variable definitions

This study used the dataset from the individual file of the 2011 Uganda AIDS Indicator Survey (UAIS 2011). The UAIS 2011 is a standardized nationally representative, population based cross-sectional survey designed to produce a range of HIV/AIDS indicators. The data has been validated and used to produce an official report [21]. The overall goal of the survey is to provide program managers and policy makers with information to effectively plan, implement and evaluate HIV/AIDS interventions. In this analysis, only women who were currently in stable marital union and whose partners had been surveyed were included. The restriction of the study sample to married and cohabiting women was necessary because only women in a stable union were asked the question regarding women’s ability to request condom use or refuse sex. This restriction also allowed for investigating the association between women’s support of MC and having circumcised partners. Though 4,900 women met the aforementioned inclusion criteria, our final sample size was 4,874 because 26 women who had never heard about HIV were excluded.

### Dependent variables

The dependent variable used for the analysis was a dichotomous indicator variable derived from the answer given to the following question: “Male circumcision is the procedure where the foreskin is removed from the penis in males. Would you recommend your male relatives/friends who are not circumcised to go for male circumcision?” This support for MC variable was coded yes or no and used as a proxy for MMC. If the respondent did not know or was unsure, it was coded no.

### Key explanatory variables

Five key independent variables were considered: (a) socioeconomic characteristics. (b) knowledge of the protective effect of circumcision, (c) having a circumcised spouse, (d) being able to request condom use, and (e) being able to refuse sex. The socioeconomic control factors in the analysis included wealth index, education level, ethnicity, age, religion, and place (rural or urban) of residence. The wealth indicators were included in the analysis as an ordinal variable of five wealth quintiles. The quintiles were computed using multiple component analysis which was based on a list of assets usually possessed in urban and rural areas of Uganda. The education variable was also an ordinal variable representing four levels of attainment: no formal education, primary school, secondary school, and tertiary school. Knowledge of the protective effect of circumcision was assessed through the answer to the question: does male circumcision help prevent getting infected with the AIDS virus? The two indicators of safer sex negotiation were derived from the women’s answers to two questions: “Can the respondent ask her partner to use a condom?” and “Can the respondent refuse sex?”.

### Other predictors variables

These included the following HIV/AIDS related variables: comprehensive knowledge of HIV/AIDS, negative attitudes toward people living with HIV (PLWHIV), and HIV testing status; these variables were all dichotomized.

### Data analyses

Descriptive statistics using number (n) and percentages (%) were used to define the characteristics of the study population. Chi-square tests were applied to assess differences in the distribution of the independent variables across the MC support categories. Bivariate and multivariate logistic regression analyses were run to assess the association between women’s support of MC and the independent factors presented above. A three-level random intercept specification was used for the multivariate analysis. This specification allowed us to account for the survey’s sampling method which is multi-clustered, with villages as the primary sampling unit selected from administrative divisions (district or regions) [22, 23]. The multilevel specification also took into account some unobservable factors at the cluster level as well as the reciprocal influence that people in the same community may have on each other. This approach has been used to model HIV testing status among Nigerian couples using the 2008 Nigeria Demographic and Health Survey (DHS) which was conducted following a sampling frame similar to the data used in this study [24]. We conducted all statistical analyses using STATA 13 and a *p*-value of ≤0.05 was used to denote statistical significance. A brief description of the model used in this study is presented below.

### Description of the three levels random intercept model

Let us denote by *y*_ijk_^*^ the latent variable representing a woman’s support for male circumcision, with i, j, k indexing respectively the woman, her village and her region.

$$ \begin{array}{c}\hfill {y}_{ijk}^{*}={a}_0+{\displaystyle \sum_{p=1}^P{\beta}_p{x}_{pijk}+{u}_{jk}+{v}_k+{\varepsilon}_{ijk}}\hfill \\ {}\hfill {u}_{jk}\sim N\left(0,{\sigma}_u^2\right)\hfill \\ {}\hfill {v}_k\sim N\left(0,{\sigma}_v^2\right)\hfill \end{array} $$

The observed response (coded as a dummy variable) is related to the latent variable by:

$$ \begin{array}{c}{y}_{ijk}=1\kern0.24em  if\kern0.24em {y}_{ijk}^{\ast }>0,\kern0.24em {y}_{ijk}=0\kern0.24em  otherwise\\ {}P\left({\varepsilon}_{ijk}<h\right)=\frac{e^h}{1+{e}^h},\kern0.48em E\left({\varepsilon}_{ijk}\right)=0,\kern0.36em var\left({\varepsilon}_{ijk}\right)=\frac{\pi^2}{3}\kern1em \end{array} $$

With *σ*_*v*_^2^, *σ*_*u*_^2^, and $\frac{\pi^2}{3}$ representing respectively the variance between regions, the variance between villages within regions, the variance between women within a village and a region by assumption. The variance between villages is equal to *σ*_*u*_^2^ + *σ*_*v*_^2^. Two different intra-class correlations for the latent responses (*y*_ijk_^*^) can also be derived from the model [25]:

Correlation across villages within the same region:

$$ {\rho}_{region}= corr\left({y}_{ijk}^{*},{y}_{i\hbox{'}j\hbox{'}k}^{*}\left|{x}_{ijk,}{x}_{i\hbox{'}j\hbox{'}k}\right.\right)=\frac{\sigma_v^2}{\sigma_v^2+{\sigma}_u^2+\frac{\pi^2}{3}} $$

Correlation across women within the same village

$$ {\rho}_{village/ region}= corr\left({y}_{ijk}^{*},{y}_{i\hbox{'}jk}^{*}\left|{x}_{ijk,}{x}_{i\hbox{'}k}\right.\right)=\frac{\sigma_u^2+{\sigma}_v^2}{\sigma_v^2+{\sigma}_u^2+\frac{\pi^2}{3}} $$

If $\rho$_*village*/*region*_ > $\rho$_*region*_, then we can suspect that women who live in the same village have similar attitudes toward male circumcision that differ from those of women who reside in the same region, but in a different village.

## Results

### Characteristics of the sample

Table 1 shows the sociodemographic characteristics of the study sample. Overall, 67.0 % (*n* = 3,276 women) of the sample were in support of MC. Most of the participants had at least primary school education (64.1 %), were less than 35 years of age (65.1 %), predominantly Catholic (42.9 %) and rural residents (86.8 %). MC supporters were more likely to be of younger age, to have at least secondary level education, of higher wealth index (*p*-value <0.0001). They were also more likely to be Muslims, urban residents and of Baganda/ Basoga ethnic groups (*p*-value <0.0001).

**Table 1:** Socio-demographic Characteristics of Sample, Uganda AIDS Indicator Survey 2011

	Support Male Circumcision			
Variables	Total (4, 874)	Yes (3,276)	No (1,598)	*P*-values
	*n* (%)	*n* (%)	*n* (%)	
Education				
No education	892 (18.3)	431 (48.3)	461 (51.7)	<0.0001
Primary	3125 (64.1)	2130 (68.2)	995 (31.8)	
Secondary	710 (14.6)	592 (83.4)	118 (16.6)	
Tertiary	147 (3.02)	123 (83.7)	24 (16.3)	
Age groups				
15–24	1356 (27.8)	956 (70.5)	400 (29.5)	<0.0001
25–34	1819 (37.3)	1267 (69.7)	552 (30.4)	
35–44	1195 (24.5)	769 (64.4)	426 (35.7)	
45 and over	504 (10.3)	284 (56.4)	220 (43.7)	
Wealth Index				<0.0001
Poorest	1032 (21.2)	511 (49.5)	521 (50.5)	
Poorer	1113 (22.8)	674 (60.6)	439 (39.4)	
Middle	985 (20.2)	682 (69.2)	303 (30.8)	
Richer	878 (18.0)	660 (75.2)	218 (24.8)	
Richest	866 (17.8)	749 (86.5)	117 (13.5)	
Religion				
Catholic	2093 (42.9)	1206 (57.6)	887 (42.4)	<0.0001
Protestant	1572 (32.3)	1069 (68.0)	503 (32.0)	
Pentecostal	396 (8.1)	263 (66.4)	133 (33.6)	
Muslim	604 (12.4)	575 (95.2)	29 (4.8)	
Other religion	209 (4.3)	163 (78.0)	46 (22.0)	
Place of Residence				
Urban	643 (13.2)	542 (84.3)	101 (15.7)	<0.0001
Rural	4231 (86.8)	2734 (64.6)	1497 (35.4)	
Access to Health facility				
Yes	1788 (36.7)	1262 (70.6)	526 (29.4)	<0.0001
No	3086 (63.3)	2014 (65.3)	1072 (34.7)	
Ethnic groups				
Baganda	596 (12.23)	523 (87.75)	73 (12.25)	<0.0001
Banyankole	402 (8.25)	274 (68.16)	128 (31.84)	
Basoga	481 (9.87)	464 (96.47)	17 (3.53)	
Bakiga	266 (5.46)	155 (58.27)	111 (41.73)	
Itesa	449 (9.21)	243 (54.12)	206 (45.88)	
Other	2680 (54.99)	1,617 (60.34)	1,063 (39.66)	

In Table 2, we present the HIV/AIDs related characteristics of the sample. About 40.2 % (*n* = 1961) of women believed that circumcision prevents HIV but only 28.0 % of the women had circumcised partners. Women who supported MC were more likely to believe that circumcision prevents HIV and were more likely to possess sexual bargaining power (i.e. refuse sex and negotiate condom use). They were also more likely to have comprehensive knowledge of HIV/AIDS and circumcised partners (*p* < 0.0001).

**Table 2:** HIV/AIDS related Characteristics of Sample, Uganda AIDS Indicator Survey 2011

	Support Male Circumcision			
Variables	Total (4,874)	Yes (3,276)	No (1,598)	*P*-values
	*n* (%)	*n* (%)	*n* (%)	
Circumcision prevents HIV				
Yes	1961 (40.2)	1738 (88.6)	223 (11.4)	<0.0001
No	2913 (59.8)	1538 (52.8)	1375 (47.2)	
Circumcised partner				
Yes	1366 (28.0)	1216 (89.0)	150 (11.0)	<0.0001
No	3508 (72.0)	2060 (58.7)	1448 (41.3)	
Request condom				
Yes	3030 (62.2)	2305 (76.1)	725 (23.9)	<0.0001
No	1844 (37.8)	971 (52.7)	873 (47.3)	
Refuse intercourse				
Yes	3762 (77.2)	2706 (71.9)	1056 (28.1)	<0.0001
No	1112 (22.8)	570 (51.3)	542 (48.7)	
Ever tested for HIV				
Yes	3743 (76.8)	2525 (67.5)	1218 (32.5)	0.506
No	1131 (23.2)	751 (66.4)	380 (33.6)	
Comprehensive HIV knowledge				
Yes	1546 (31.7)	1181 (76.4)	365 (23.6)	<0.0001
No	3328 (68.3)	2095 (62.9)	1233 (37.1)	
Stigmatize PLHV				
Yes	3807 (78.1)	2610 (68.6)	1197 (31.4)	0.0002
No	1067 (21.9)	666 (62.4)	401 (37.6)	

### Factors associated with women’s support of male circumcision

In Table 3, we illustrate the results from the bivariate and the multivariate logistic regression analyses. The likelihood ratio test, which compares a simple logistic specification to that of the three-level random intercept, showed that the latter represents the better fit with the data (chi-square = 460, *p* value < 0.0001). The analysis of the intra-class correlation revealed that the within-region (between villages) difference accounted for 29 % (ICC = 0.29, 95 % CI: 0.18, 0.43) of the total variance, while 18 % (ICC = 0.18, 95 % CI: 0.08, 0.37) of the total variance stemmed from differences between regions. This result suggests that the attitudes of women from the same village were correlated.

**Table 3:** Predictors of Married Women’s Support of Male Circumcision in Uganda

	Crude OR (95 % CI)	AOR (95 % CI)
Circumcision prevent HIV	6.97 (5.95–8.15)***	5.85 (4.83–7.1)***
Circumcised male partner	5.70 (4.75–6.84)***	3.29 (2.49–4.37)***
Request condom use	2.86 (2.53–3.24)***	1.79 (1.48–2.16)***
Refuse sexual intercourse	2.44 (2.12–2.80)***	1.45 (1.18–1.77)***
Ever tested for HIV	1.05 (0.91–1.21)	1.11 (0.90–1.37)
Comprehensive HIV	1.90 (1.66–2.18)***	1.30 (1.08–1.58)**
Stigmatize PLHV	1.31 (1.14–1.51)***	0.93 (0.76–1.14)
Education		
No education	1.00	1.00
Primary	2.29 (1.97–2.66)***	1.46 (1.17–1.82)***
Secondary	5.37 (4.23–6.80)***	1.41 (0.99–2.01)
Tertiary	5.48 (3.47–8.65)***	1.31 (0.70–2.46)
Age group		
15–24	1.00	1.00
25–34	0.96 (0.82–1.12)	1.23 (0.99–1.51)
35–44	0.76 (0.64–0.89)***	1.08 (0.85–1.37)
45 and over	0.54 (0.44–0.67)***	0.91 (0.67–1.23)
Wealth quintiles		
Poorest	1.00	1.00
Poorer	1.57 (1.32–1.86)***	1.07 (0.85–1.36)
Middle	2.29 (1.91–2.75)***	1.07 (0.82–1.39)
Richer	3.09 (2.54–3.75)***	1.32 (0.99–1.77)
Richest	6.53 (5.19–8.21)***	1.74 (1.15–2.62)**
Religion		
Catholic	1.00	1.00
Protestant	1.56 (1.36–1.79)***	1.07 (0.88–1.30)
Pentecostal	1.45 (1.16–1.82)**	0.97 (0.71–1.32)
Muslim	14.58 (9.94–21.39)***	3.81 (2.32–6.28)***
Other religion	2.61 (1.86–3.66)***	1.06 (0.67–1.66)
Residence urban/rural	2.94 (2.35–3.67)***	0.99 (0.61–1.61)
Access to health facility	1.28 (1.13–1.45)***	1.08 (0.89–1.31)
Ethnic groups		
Baganda	1.00	1.00
Banyankole	0.30 (0.22–0.41)***	1.02 (0.602–1.744)
Basoga	3.81 (2.21–6.55)***	1.96 (0.960–4.017)
Bakiga	0.19 (0.14–0.28)***	0.76 (0.435–1.322)
Itesa	0.16 (0.12–0.22)***	0.52 (0.307–0.898)*
Other group	0.21 (0.16–0.27)***	0.77 (0.517–1.160)
Intraclass correlation		
Primary sampling unit		0.29 (0.18–0.43)***
Region		0.18 (0.081–0.37)**

Significance of *p*-values: ***p < 0.001, **p < 0.01, *p < 0.05

*OR* odds ratio, *AOR* adjusted odds ratio

Younger, wealthier, more educated, and Muslim women were more likely to support MC. Religion was one the greatest predictor of MC as Muslims were about four times as likely to support MC compared to Catholics (AOR = 3.81, 95 % CI 2.32–6.28). With regards to ethnicity, women of Itesa ethnic group were about 50 % less likely than those of Baganda ethnic group to support MC (AOR = 0.52, 95 % CI 0.307–0.898). Women who knew that MC reduces the risk of HIV transmission were more likely to support MC (AOR = 5.85, 95 % CI 4.80–7.1). Comprehensive knowledge of HIV/AIDS was positively associated with women’s support of MC (AOR = 1.30, 95 % CI 1.00–1.58). Women who had a circumcised partner were 3.3 times as likely to support MC compared to their counterparts whose partners were not circumcised (AOR = 3.29, 95 % CI 2.49–4.37). The two indicators of women’s sexual bargaining power were positive predictors of support for MC. Women who could negotiate condom use were almost twice as likely to support MC (AOR=1.79, 95 % CI 1.48–2.16), and women who reported having the ability to refuse sex were about 50 % more likely than their counterparts to support MC (AOR 1.45, 95 % CI 1.18–1.77).

## Discussion

This study investigated factors that influence women’s support of MC in Uganda. The results show that about 67 % of married women supported MC; that is, they were willing to recommend MC to their male relatives. This is consistent to some extent with findings from previous studies [13, 26]. Although Westerkamp and Baily [13] included all women from diverse countries, irrespective of their marital status, our findings do share a significant overlap in consistency with theirs. Similarly, Mavhu et al.[26] found that 58 % and 60 % of women in their study (which include 67 % of married women) in rural Zimbabwe would respectively like their partners and their sons circumcised if it protected them against HIV. The restriction of the sample used for our analysis to only married women could explain the slightly higher prevalence of MC support in our study. Though we found high level of support for MC only about one–third of married women who supported MC had circumcised partners. This discordance might be due to the fact that MMC scale–up is a new idea in Uganda [3] and the translation of increase in women’s support and recommendation to uptake of circumcision will take a while. Other barriers to circumcision such as lack of knowledge of health unit where circumcision can be performed, cost, disbelief that MC protects against HIV, cultural issues, fear of pain or adverse effect [18, 26, 27] may also explain this low level of partner’s circumcision rate in the presence of high level of women’s support. The greatest positive predictors of married women’s support of MC in our study were knowledge that MMC can decrease the risk of HIV transmission, having a circumcised male partner, being Muslim and women’s sexual bargaining power (measured by ability to request condom use and refuse sexual intercourse). An innovative aspect of this research is our investigation of the role of women’s sexual bargaining power in women’s support of MC.

The positive association between knowledge that MC reduces risk of HIV infection and women’s support of MC corroborates findings from other studies conducted in East African nations [16, 26]. Our findings also demonstrated that wealthier women are more likely to support MC than their counterparts with lower wealth status. The positive correlation between education and women’s support of MC is consistent with the result of studies conducted in Kenya [18, 26]. The association between having circumcised partners and married women’s support of MC could be explained by the role of other predictors of women’s support of MC. These include the benefits of circumcision such as the potential increase of pleasure [18, 28] and penile hygiene [10, 29]. We also observed a positive association between urban residency and women’s support of MC in the unadjusted model. This concurs with findings from the KwaZulu–Natal province in South Africa [28]. However, the fact that the association became insignificant in the multivariate model suggests that it is not urban residency per se that facilitates women’s support of MC but rather the preponderance of other facilitators in the urban area. These facilitators may include higher wealth level, knowledge and the reduction of some ethnicity barriers.

Religion and ethnicity were also associated with MC support in this study. Married Muslim women were more likely than Catholics to support MC. This is because Muslims practice male circumcision for religious reasons [30]. However, unlike other public health topics where religious doctrine is against a health practice (such as the Catholicism and the use of modern contraception), there is no religious doctrine that prohibits male circumcision [31]. With regards to ethnicity, women of Itesa ethnic group were less likely than those of Baganda ethnic group to support MC. Report from the 2011 Demographic and Health Survey showed that men of Itesa ethnic group had the lowest prevalence of circumcision (7 %) in Uganda [32]. Further research is required to understand why Itesa ethnic group women do not support MC and the reason for low circumcision rate among their male population. Targeted intervention is required to increase MC support among sub–populations such as Catholics, other Christians, and the Itesa ethnic group.

Based on the findings from the random effects test, our study also revealed that significant variations exist in women’s support of MC across regions in Uganda. That the intra–class correlation remained significant after controlling for ethnicity and religions signifies that other unobservable influential factors may exist and remain unaccounted for. Further research is required to explore the reasons for the differences in women’s support of MC across regions in Uganda.

Our study has some limitations. First, the variable male circumcision (MC) was used as a proxy for medical male circumcision (MMC). This could have resulted in an overestimation of support for MMC and the measures of association for MMC support. Second, due to the structure of the dataset, our sample was restricted to only married women. Therefore, our findings must be interpreted with caution as factors associated with MC support among unmarried younger women in uncommitted relationships may be different than those for married women. For instance, the effect of sexual bargaining power on MC support may be different for this population and so interventions to improve their sexual bargaining power may also be unique. Further research is required to explore factors associated with support of MC among this population and whether their support actually influence circumcision uptake.

The strengths of this study include the large sample size and generalizability of study findings to married women in Uganda due to the use of a nationally representative dataset. The outcome, support for MC was based on a very sensitive query that correctly classifies individuals who support and do not support MMC: ‘Would you recommend your male relatives/friends who are not circumcised to go for male circumcision’. This is because if an individual recommends MC, it is highly probable they will support MS. The converse is also true. To our knowledge, this is the first to demonstrate a relationship between women’s sexual bargaining power and support for MC.

## Conclusion

This study has identified several factors that may be targeted in order to increase uptake of MMC among Ugandan men. The importance of promoting educational campaigns to increase women’s knowledge of the benefits of MMC is key. Tailored interventions toward women irrespective of their age, particularly poor women and those of religious groups that are less likely to support MMC is recommended. It is also essential to develop programs and policies that can equip married women with the skills and/or power to negotiate safer sex. Overall, this study underscores the necessity to give prime importance to the role of women and the need to include them in interventions that aim at achieving MMC scale–up in Uganda.

## Abbreviations

AIDS, Acquired immune deficiency syndrome; CI, Confidence intervals; HIV, human immunodeficiency virus; MMC, medical male circumcision, MC male circumcision; PEPFAR, President’s Emergency Plan for AIDS Relief; UNAIDS, United Nation Joint Program on HIV/AIDS; WHO, World Health Organization
